# Draft Genome Sequence of *Bacillus coagulans* NL01, a Wonderful l-Lactic Acid Producer

**DOI:** 10.1128/genomeA.00635-15

**Published:** 2015-06-18

**Authors:** Zhaojuan Zheng, Ting Jiang, Xi Lin, Jie Zhou, Jia Ouyang

**Affiliations:** a College of Chemical Engineering, Nanjing Forestry University, Nanjing, People’s Republic of China; b Jiangsu Key Lab of Biomass-based Green Fuels and Chemicals, Nanjing, People’s Republic of China; c Key Laboratory of Forest Genetics & Biotechnology of the Ministry of Education, Nanjing, People’s Republic of China

## Abstract

Here, we report the draft genome sequence of *Bacillus coagulans* NL01, which could produce high optically pure l-lactic acid using xylose as a sole carbon source. The draft genome is 3,505,081 bp, with 144 contigs. About 3,903 protein-coding genes and 92 rRNAs are predicted from this assembly.

## GENOME ANNOUNCEMENT

*Bacillus coagulans* is a wonderful l-lactic acid producer, which is also thought to be a promising producer for high-value chemicals. However, little is known about this species (1). Recently, there has been an extraordinary accumulation of knowledge on the genomics of these organisms. *B. coagulans* NL01 was isolated from soil using a medium with xylose as the sole carbon source (2). In our previous studies, a maximum lactic acid concentration of 75.0 g/liter was achieved from xylose by *B. coagulans* NL01 in batch fermentation. Moreover, this strain also exhibited high fermentation ability from low-cost lignocellulosic feedstocks (2–4). With glucose-rich lignocellulosic hydrolyzate as a substrate, 75.0 g/liter lactic acid was produced (4). When using xylose-rich corn stove prohydrolysate as a substrate, 23.5 g/liter lactic acid was obtained in 36 h (3). To better understand the fermentation process of *B. coagulans* NL01, especially for xylose fermentation, the genome of *B. coagulans* NL01 was sequenced.

The genomic DNA of strain NL01 was extracted using a bacterial DNA kit (Omega). Its quality and quantity were examined and measured using a NanoDrop2000 spectrophotometer (Thermo Scientiﬁc) and the Quant-iT PicoGreen double-stranded DNA kit (Invitrogen). Whole-genome sequencing was performed using Illumina MiSeq and generated 1,578,349 paired-end reads of 300 bp with 200-fold coverage on average. Paired-end reads were merged by FLASH (5), and the long-reads were assembled *de novo* into contigs using Newbler (6). The draft genome yields 144 contigs with a G+C content of 46.23% and a total assembly length of 3,505,081 bp. The annotation of the genome was performed using RAST (http://rast.nmpdr.org) (7).

Annotation in RAST revealed 92 RNAs, 3,903 coding sequences, and 406 subsystems. There were 450 genes involved in carbohydrates, which contain all the necessary genes for trichloroacetic acid (TCA) and lactic acid production. Also, three l-lactate dehydrogenases and one lactate permease were predicted by RAST. RAST also identified three genes involved in the subsystems of xylose utilization, including xylose isomerase, xylulose kinase, and a repressor, open reading frame, kinase (ROK)-family xylose-responsive transcription regulator. Little genetic information was found by RAST, which may be due to the lack of knowledge about this strain. In addition to the lactic acid production, genes required for the production of high-value chemicals (acetoin and butanediol) were found in the annotation, which implies the potential application as a biocatalyst for high-value chemicals. Like other *B. coagulans* strains (1), we also identified the genes involved in the clusters of regularly interspaced short palindromic repeat (CRISPR)-cas systems, such as *cas1-3*, *cas5*, and *cas6*. The CRISPR-cas system was believed to act as a bacterial immune system to keep the host from phage infection. In-depth comparative analysis among different species is now the focus of our work.

### Nucleotide sequence accession numbers. 

This whole-genome shotgun project has been deposited at DDBJ/EMBL/GenBank under the accession no. LBMQ00000000. The version described in this paper is version LBMQ01000000.
